# Fibrodysplasia ossificans progressiva—a rare disease with distinctive features yet still a diagnostic challenge: A case report

**DOI:** 10.1097/MD.0000000000019933

**Published:** 2020-04-24

**Authors:** Xiaofei Shi, Liqing Zhou, Jingjing Shang, Ke Wang, Cong-Qiu Chu

**Affiliations:** a Department of Rheumatology and Immunology, The First Affiliated Hospital and College of Clinical Medicine, Henan University of Science and Technology, Luoyang, China; b Division of Arthritis and Rheumatic Diseases, Oregon Health & Science University and VA Portland Health Care System, Portland, OR.

**Keywords:** fibrodysplasia ossificans progressiva, heterotopic ossification, activin receptor 1A

## Abstract

**Rationale:**

Fibrodysplasia ossificans progressiva (FOP) is rare genetic disease featuring progressive heterotopic ossification of soft tissues of the musculoskeletal system which leads to severe disability and premature death. Recognition of this disease is important since invasive diagnostic procedures can promote disease progression. However, despite its distinctive clinical manifestations, diagnosis can be difficult because of its rarity

**Patient concerns:**

A 20-year-old woman was referred to rheumatology clinic for management of “ankylosing spondylitis”. The patent had begun to have hard subcutaneous nodules when she was 1 year old, and subsequently developed hip joint pain and flexion contractures of knees and hips leading to disability.

**Diagnoses:**

Based on characteristic bilateral great toe deformities and radiographic images of ossification of soft tissues, a clinical diagnosis of FOP was made. This was confirmed by genetic test showing a heterozygous mutation (c.G617A) of the activin receptor 1A gene (*ACVR1*).

**Interventions:**

The patient was treated symptomatically and with supportive measures, and her condition remained stable.

**Lessons:**

Diagnosis of FOP can be difficult, despite its distinctive clinical manifestations, because of its rarity. Recognition of this disease is important to avoid invasive diagnostic procedures which can promote progression.

## 1 Introduction

Fibrodysplasia ossificans progressiva (FOP), also known as myositis ossificans progressiva (MOP), is an extremely rare genetic condition causing heterotopic ossification of the musculoskeletal system. The estimated prevalence is 1 in 2 million with no predisposition by race, gender or geographical distribution.^[1]^ FOP is caused by a heterozygous missense mutation (c.617G > A) of the gene encoding activin receptor IA (ACVR1). ACVR1 is a bone morphogenetic protein (BMP) type I receptor. The majority of FOP cases are sporadic de novo mutations.^[1]^ Genetic transmission, when observed, is autosomal dominant. The *ACVR1* mutation results in overactive BMP signaling which leads to ectopic osteognesis of soft tissue. Clinically, FOP is characterized by progressive heterotopic ossification of the striated muscle, tendon, ligament, fascia, aponeurosis, and skin^[2,3]^ which leads to permanent disability. Because of its rarity, the diagnosis of FOP can be challenging.^[4]^

Patient has provided informed consent for publication of the case.

## 2 Case presentation

A 20-year-old female was referred to our rheumatology clinic by her primary care physician for management of severe “ankylosing spondylitis”. She had left hip pain for 8 years, and gradually developed severely limited range of motion and rigidity of that hip. Subsequently, her left knee, both shoulders, and both ankles had intermittent swelling with mild pain, and limitation in range of motion progressed. Her mother noticed that the range of motion in the cervical and lumbar spine has been progressively limited gradually over the last 8 years. She also had difficulty in opening her mouth for the last 3 years. These symptoms were partially improved by naproxen or Ibuprofen, which she took as needed, but limited range of motion of her joints progressed and she had become wheelchair bound. One week before the presentation to our clinic, her right elbow was severely swollen and painful, with limited range of motion. Her mother noticed intermittent subcutaneous nodules since the daughter was 1 year old. The nodules presented on the trunk or extremities, often around the shoulders, elbows, and hips. They typically appeared as hard, non-tender and non-painful masses. They did not affect her daily school and physical activities; and most resolved spontaneously, though some become persistent. The parents did not seek medical care since these symptoms did not affect her growth or activities. She has no family history of a similar condition.

**Figure 1:**
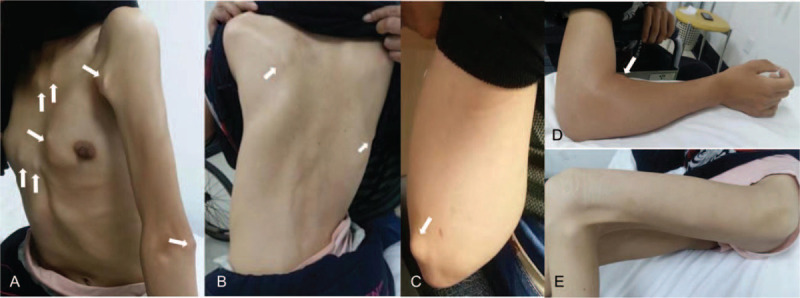
Musculoskeletal manifestations. (A–D) There are multiple hard subcutaneous nodules about 1 x 1 cm in size (white arrow). (C) Swelling of right elbow with mild tenderness but without increased warmth of the skin. (E) Flexion contracture of left hip and knee.

**Figure 2:**
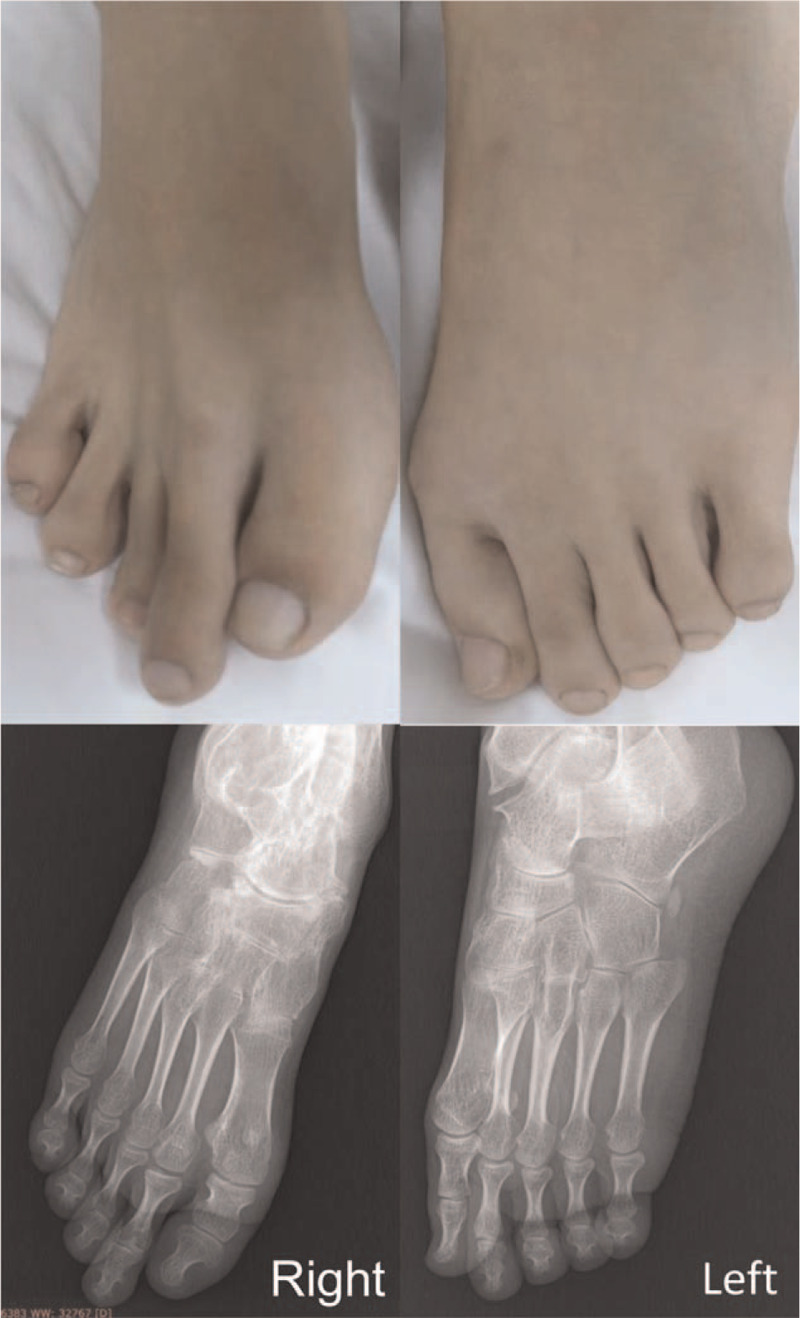
Shortness of the great toe of right foot.

On physical examination, she appeared thin without distress, and ambulated with a crutch. Her maximum oral aperture was limited. There were multiple subcutaneous nodules on the posterior and anterior chest wall, around the shoulders and upper and lower extremities. The nodules were approximately 1 x 1 cm in size, irregular, hard, immovable, and non-tender. There was no increased warmth or erythema of the overlying skin (Fig. 1). The cervical spine had severely limited range of motion in all directions. The thoracic and lumbar spines were scoliotic (Fig. 1) with limited flexion and extension. There was muscle atrophy which was more severe in the lower extremities. The right elbow joint was swollen, mildly tender, with limited range of motion (Fig. 1). The left hip and left knee had flexion contractures (Fig. 1). Shoulders and ankles had limited range of motion bilaterally, but less severe and had no swelling. Her great toes were shorter than the other toes (Fig. 2).

**Figure 3:**
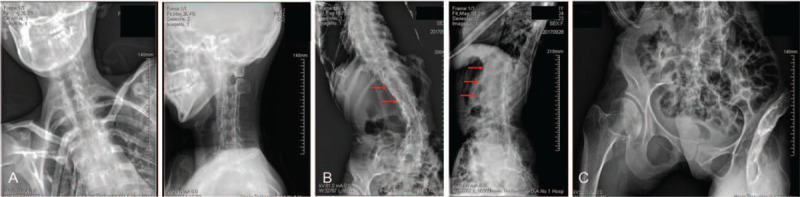
Radiographs of spine and sacroiliac joints. (A) Loss of physiological curvature of the cercal spine. (B) Kyphoscoliosis of thoracic and lumbar spines with ossification of the anterior longitudinal ligament. (C) Fusion of bilateral sacroiliac joints.

**Figure 4:**
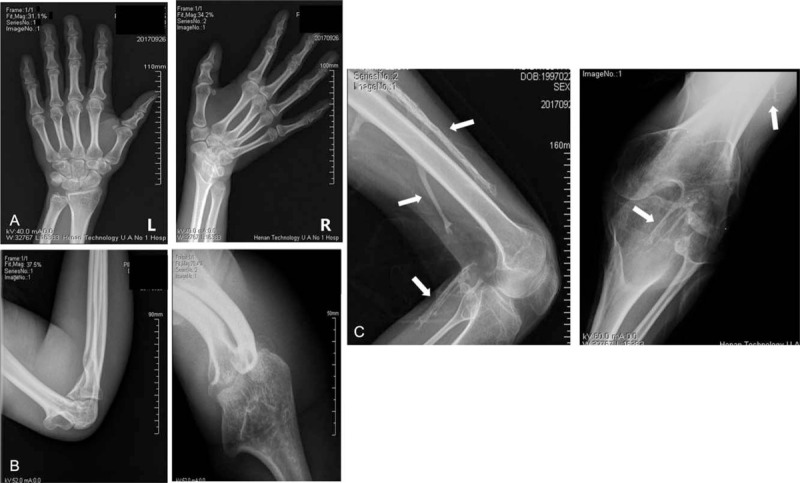
Heterotopic ossification. (A) Fusion of bilateral fifth distal interphalangeal joints. (B) Right elbow joint with expansion of the distal humerus, uneven bone density, fusion of the radio-humeral joint and narrowing of the ulno-humeral joint. (C) Flexion contracture of left knee and multiple linear densities of varying length in the subcutaneous tissues around the knee, most aligned with the long axis of the femur (white arrows).

Radiographs of the spine showed loss of the physiological curvature of the cervical spine, and thoracic and lumbar kyphoscoliosis. The vertebral body showed square changes and multiple inter vertebral space narrowing. Anterior longitudinal ligament ossification was apparent in the cervical spine, and more marked in the thoracic and lumbar spine with marginal bridging (Fig. 3). The facet joints showed ossification. Bilateral sacroiliac joint fused (Fig. 3). Radiographs of peripheral joints showed fusion of the bilateral fifth distal interphalangeal joints (Fig. 4); expansion of the distal humerus with irregular bone density, fusion of the radial-humeral joint and narrowing of the ulno-humeral joint space (Fig. 4). The most striking radiographic changes were at the left knee and surrounding soft tissue. The joint space was markedly narrowed and the joint remained flexed at 90 degrees. Multiple bone density stick-like structures of varying length were present around the knee and most of which were along the long axis of the femur (Fig. 4) indicating ossifications of soft tissues.

Blood tests showed normal erythrocyte sedimentation rate and C-reactive protein. Anti-nuclear antibodies, rheumatoid factor, anti-cyclic citrullinated peptide antibodies, and HLA-B27 were all negative. Serum creatine phosphokinase, aldolase, calcium, phosphorus, alkaline phosphatase, and parathyroid hormone levels were all normal.

**Figure 5:**
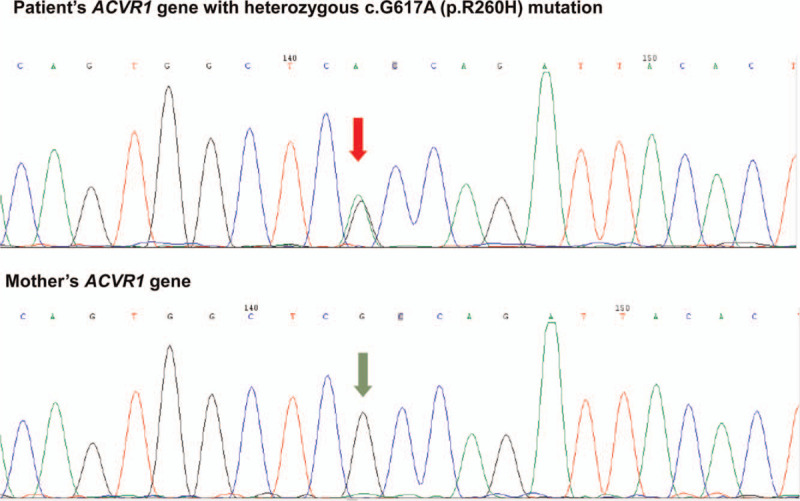
Heterozygous mutation of *ACVR1* gene (cG617A) of the patient (top panel) and no ACVR1 gene mutation of patient's mother (bottom panel).

Based on the history, typical findings of short great toes, and striking radiographic images, FOP was suspected and was subsequently confirmed by the presence of a heterozygous c.G617A (p. R206H) mutation of the *ACVR1* gene (Fig. 5).

Symptomatic treatment with ibuprofen or naproxen as needed was “offered”. Bisphosphonates were prescribed but discontinued due to gastrointestinal intolerance. Physical therapy was focused on maintaining the residual range of motion of her joints. Her physical disability has shown no significant progression at 1 year of follow up.

## 3 Discussion

FOP is characterized by abnormal ossification in muscles, ligaments, tendons, and joint capsules. Symptoms usually develop by 10 years of age, with an average age of onset of 7.1 years.^[5]^ There is no obvious precipitating event which can be tracked to FOP onset. The first symptoms are recurring soft tissue masses, which can become small or subside. The residual mass will then harden to form an ectopic ossification block. Additional masses subsequently form in new locations. FOP patients usually have the great toe deformity at birth as seen in this case (Fig. 3), a feature that contributes to the differential diagnosis of FOP and other bone and muscle diseases.^[6]^ Other common features include proximal medial tibial osteochondroma, in situ fusion of the posterior component of the cervical spine, a broad short femoral neck and conductive hearing loss. Lesions usually cause abnormalities in the tongue, diaphragm, and extraocular muscles, but do not involve smooth muscles or myocardium.^[4,5]^ The ossification in patients with FOP progresses characteristically downward from the top of the body, similar to the bone development of the fetus. The patient usually begins to ossify in the neck, then the shoulders, arms, chest area, and finally the feet. Specifically, ossification in the soft tissues is typically first seen in the back, skull, and proximal regions, then progresses to involve the abdomen, limbs and distal areas. However, due to unexpected events such as injuries, disease progression does not necessarily occur in this order.^[5]^

Despite the distinctive clinical features, about 90% of FOP cases are misdiagnosed, and 67% undergo unnecessary, harmful invasive diagnostic procedures.^[4,7,8]^ In a largest published series including 72 cases of FOP, 84% of the patients were misdiagnosed or under-diagnosed,^[4]^ as in our patient who was referred to rheumatology clinic for management of “ankylosing spondylitis”. Indeed, some features of her radiographic findings in the spine and sacroiliac joints could be misinterpreted as features of ankylosing spondylitis. The main reason for misdiagnosis is lack of awareness of the disease because of its rarity. In this case series, 36% patients underwent invasive biopsy procedures which led to the development of heterotopic ossification at the operative sites. Therefore, invasive diagnostic procedure or surgeries should be avoided to minimize the potential risk to promote the progression of FOP.^[4,7,8]^ At present, the diagnosis of FOP mainly relies on the clinical manifestations of patients, including early multiple soft tissue nodules, progressive heterotopic ossification and congenital malformation of the big toe. Plain X-ray and computed tomography can show heterotopic ossification, which helps to better define the lesions.^[9]^ Premalignant ossification of diffuse soft tissues on magnetic resonance imaging early in the disease provides an important clue for early diagnosis of FOP.^[10]^

The underlying genetic defect in FOP is a missense mutation of *ACVR1* gene, c.617G>A (R206H). This mutation leads to the loss of stability of the glycine-serine region, which causes continuous activation of ACVR1 and leads to ectopic cartilage osteogenesis and joint fusion in FOP patients. Although FOP can be inherited via an autosomal dominant mode,^[11]^ most of the cases reported are caused by spontaneous mutations.^[1]^ Moreover, in a 72 case series, all of the cases are sporadic.^[4]^ Therefore, in a typical clinical case, a negative family history of FOP does not preclude the diagnosis. It is highly likely that our case was caused by a spontaneous mutation. This is affirmed by her negative family history and negative genetic test in her mother. Unfortunately, we were not able to obtain genetic testing for her father, though he does not display any clinical manifestations similar to those presented in our patient. Awareness of the disease is the key factor for making an accurate diagnosis of FOP.

Management of FOP is challenging since there is no effective, targeted therapy. The average of life expectancy of patients with FOP is about 40 years, and most patients died due to pulmonary complications.^[12]^ The goal of treatment is palliative pain relief and preservation of musculoskeletal function.^[12]^ The mainstay of non-interventional treatment consists of nonsteroidal anti-inflammatory drugs and physical therapy with various modalities. Diaphragmatic breathing and inspiratory resistance exercises may improve respiratory function. Short-term use of corticosteroids for relief of flare-ups is effective,^[13]^ but is not recommended for long-term use. In a clinical trial including 5 FOP patients who were treated with perhexiline maleate for 12 months and followed up for 2 years, no significant benefit was observed.^[14]^ Surgical intervention is generally to be avoided but may be applied in selective cases to remove painful masses or correction of deformities.^[12,15]^ Radiation therapy following surgical removal of mass has been applied^[16]^ and was used in prevention of rapid deterioration of ambulation level.^[17]^ Since discovery of the determinant gene, targeted therapies to inhibit heterotopic ossification by acting on different pathways, including infusion of anti-activin A antibody, have been evaluated in vitro and in mouse models^[18,19]^ providing hope for better future management of FOP.

In summary, FOP is an extremely rare disease which is often misdiagnosed in spite of its distinctive features. Awareness of this condition is key for making the diagnosis and avoiding unnecessary invasive diagnostic procedures which may promote progression. Currently management of FOP is mainly symptomatic relief and maintenance of physical function. Ongoing research to develop effective therapies to halt progression of the disease by targeting the ACVR1 signaling pathway is promising.

## Acknowledgments

The authors would like to thank Dr. Leslie Kahl for her editing and review of the manuscript.

## Author contributions

**Conceptualization:** Cong-Qiu Chu, Xiaofei Shi.

**Data curation:** Xiaofei Shi, Liqing Zhou, Jingjing Shang, Ke Wang.

**Investigation:** Xiaofei Shi, Liqing Zhou, Jingjing Shang, Ke Wang.

**Methodology:** Xiaofei Shi, Liqing Zhou.

**Supervision:** Cong-Qiu Chu.

**Writing – original draft:** Cong-Qiu Chu, Xiaofei Shi, Ke Wang.

**Writing – review & editing:** Cong-Qiu Chu, Xiaofei Shi, Liqing Zhou, Jingjing Shang, Ke Wang.
